# Understanding Violent Behavior in Early Psychosis Through the Fluid Vulnerability Theory: an Exploratory Qualitative Study

**DOI:** 10.1007/s10597-025-01504-6

**Published:** 2025-09-09

**Authors:** Stephanie A Rolin, Megan G Flores, Deirdre Caffrey, Jennifer Mootz, Lisa B Dixon, Paul S Appelbaum, Barbara Stanley, Leah G Pope

**Affiliations:** 1 Columbia University Irving Medical Center, New York, USA; 2 Department of Psychiatry and Behavioral Sciences, Howard University, Washington, DC USA

**Keywords:** Psychosis, Violence, Early intervention, Suicide, Qualitative

## Abstract

Compared to the general population, young adults with early psychosis are at increased risk of violent behavior. Existing research has found contextual similarities between violent behavior and suicidal behavior. Therefore, this study examines the drivers and consequences of violent ideation and behavior among young adults with early psychosis by applying frameworks developed for suicide prevention. This research was conducted at OnTrackNY, a network of early intervention services (EIS) that provides coordinated specialty care services to young adults with non-affective psychosis that began within the past two years. Qualitative interviews were conducted with 6 EIS participants and 12 EIS staff members. The interview guide applied the “suicide narrative” format from the Stanley-Brown Safety Planning Intervention by asking EIS staff and EIS participants detailed questions about a specific episode of violent ideation or behavior. Participant responses were then categorized using Fluid Vulnerability Theory (FVT). This study found that it was feasible to discuss information about a specific violence-related crisis for young adults with early psychosis and their treatment teams by eliciting violence narratives. In doing so, EIS participants and staff identified several risk factors from the FVT domains that previously sparked either violent ideation or behavior. In addition to identifying potential target mechanisms for future interventions, these narratives may lead to more compassionate and therapeutic understandings of violence for young adults with early psychosis. Future research is recommended to explore how best to incorporate violence narratives in the treatment of early psychosis.

## Introduction

Current research estimates that approximately one-third of young adults with early psychosis engage in violence during the early course of their illness (Rolin et al., 2019). Prompt treatment during this period has led to improvements in outcomes, such as greater involvement in work or school, reduced symptom severity, and higher satisfaction with treatment (Fusar-Poli et al., 2017). Yet, no evidence-based interventions to reduce violence have been developed and tested among young adults with early psychosis (Rolin et al., 2024a, b).

In contrast, several suicide prevention interventions have been developed and tested for young adults with early psychosis (Stanley & Brown, 2019). Although violent and suicidal behaviors differ in important ways, they also share notable similarities, such as the mechanisms of anger, impulsivity, and emotional dysregulation (Ammerman et al., 2015). Both suicidal behavior and violent behavior can have severe health consequences. Existing public health research has found that people being exposed to suicidal behavior by someone they know can lead to lower overall well-being and increased suicidal ideation, while exposure to violent behavior has been linked to increased risk of depression, substance use, and suicidal behavior (Farrell & Zimmerman, 2019; Huang et al., 2018; Hvidkjaer et al., 2021). Additionally, both exposure to violence in childhood and violent behavior in adulthood are risk factors for suicidal behavior (Jokinen et al., 2010).

Given these similarities, this research seeks to use “violence narratives” (analogous to the “suicide narrative” format from the Stanley-Brown Safety Planning Intervention) to elucidate descriptions of violent episodes. We will apply the Fluid Vulnerability Theory (FVT), a well-known theoretical framework developed in suicide research, to analyze participant responses. FVT hypothesizes that the state of suicidality and its triggering factors are fluid in nature and duration. Similarly, research regarding violence among persons with psychosis has found that violence risk comprises both static and dynamic risk factors (Purcell, 2015). The FVT also states that all persons have a baseline level of suicide risk that is predicted by historical and developmental factors, and a threshold at which “the suicidal mode” (i.e., a state of mind in which a person is more likely to act on suicidal thoughts) is activated. (Rudd, 2006). This is comparable to how violence risk is assessed in clinical settings via a combination of historical factors (such as a history of violence, or hallucinations or delusions with violent content) and dynamic factors (such as treatment instability or substance use) (Purcell, 2015). Under the FVT, suicide risk can be increased by internal or external aggravating factors that fall into one of four domains: (1) the cognitive system, (2) the affective system, (3) the physiological system, and (4) the behavioral (motivation) system (Rudd, 2006). This research will analyze “violence narratives” through the lens of the FVT, sorting internal or external aggravating factors into these four domains. This approach is selected given the need for evidence-based interventions to reduce violence, and the utility of the FVT in guiding intervention development to reduce suicide risk for diverse populations with a variety of mental health diagnoses (Rugo-Cook et al., 2021; Wolford-Clevenger & Smith, 2017). Therefore, the aims of this study are (1) to assess the feasibility of utilizing a narrative format to elicit lived experiences of violent ideation or behavior and (2) understand these narratives/lived experience through the framework of FVT.

## Methods

### Setting

This study was conducted within the OnTrackNY network, an evidence-based model of early psychosis care that provides early intervention services (EIS) with 28 locations in New York State. Each clinic provides coordinated specialty care services to young adults fulfilling the following criteria for treatment: (1) 16–30 years old; (2) diagnosis of a non-affective psychosis and a history of psychotic symptoms lasting at least one week; (3) first onset of psychotic symptoms in the past two years; and (4) a resident of New York State. While participants were enrolled based on a diagnosis of non-affective psychosis, some exhibited affective symptoms such as mania during treatment. This reflects the real-world overlap of mood and psychotic symptoms commonly seen in EIS programs (Moran, 2019). Full details about the OnTrackNY program, including detailed inclusion criteria, exclusion criteria, and operational details are described in previous publications (Bello et al., 2017; Dixon et al., 2015; Rolin et al., 2024a, b).

### Study Design

This study was embedded within a parent study assessing the potential feasibility and acceptability of a behavioral intervention to reduce violence for young adults with early psychosis enrolled in EIS (Rolin et al., 2024a, b). To achieve this aim, qualitative interviews were conducted with EIS staff (clinicians and peer specialists) and EIS participants. Each interview lasted approximately one hour and was conducted in-person for EIS participants, and either in-person or remotely (via Zoom^®^) for EIS staff.

### Participants

EIS staff (licensed clinicians and peer specialists) were recruited through an email sent to position-specific listservs of current OnTrackNY staff, with the support of an OnTrackNY Stakeholders Workgroup. Interested EIS staff contacted the research team to set up an interview time and provide documented informed consent via Zoom. Eligibility criteria for EIS staff included prior experience treating EIS participants with violent behavior in an OnTrackNY clinic. This study’s sample size was based on the needs of the parent study. In that study, recruitment ended after 12 EIS staff interviews and 6 EIS participant interviews due to data saturation (Rolin et al., 2024a, b).

This study interviewed EIS participants with violent behavior or ideation in the past six months. Both behavior and ideation were included to facilitate the development of proactive responses to violence. EIS participants were recruited using purposive sampling from one OnTrackNY clinic, having been referred to the study team by their EIS clinician. All interactions with EIS participants happened in-person at their OnTrackNY clinic. Participants were screened for violent ideation or violent behavior using the Columbia Initial Screen for Violence (CIS-V), which screens for ideation or behavior such as: minor violence not causing physical injury; acts of battery causing physical injury; sexual assault; assaultive acts involving the use of a weapon; or threats made with a weapon in hand (Masucci et al., n.d.). Because the focus of this study is to address psychosis-related violence, potential EIS participants were also screened for premorbid histories of antisocial behavior prior to age 15 with the *Structured Clinical Interview for Axes I and II DSM-IV Disorders* (First et al., 2015). This differentiation is based on a post-hoc analysis from the Clinical Antipsychotic Trials of Intervention Effectiveness (CATIE) study, which distinguished participants with psychosis-related violence from those with violence related to pre-existing anti-social behavior (Swanson et al., 2008). Persons with antisocial behavior scores above the median of the CATIE study sample were excluded, based on existing research recommending separate treatment strategies for addressing antisocial-related violence (Adams & Yanos, 2020; Moran & Hodgins, 2004; Mueser et al., 2006; Swanson et al., 2008; Volavka & Citrome, 2011).

All interviews were completed by 2 female physicians with training in qualitative research techniques (SAR, a board eligible psychiatrist and DC, a psychiatry resident). Interviews were 30–60 min long. No prior relationship existed between the researcher and participants before the start of the study and no one else was present during the interviews. No repeat interviews were conducted. Participants were informed of the researcher’s name, institutional affiliation, and the purpose of the study. The interviewers maintained a neutral stance during data collection; no personal biases or assumptions were shared with participants. Minimal field notes were recorded informally to capture logistical details and general reflections; transcripts were not shared with participants for comment and participants did not provide feedback on the findings.

Of the eight prospective EIS participants referred to the study team, one participant was ineligible (due to having no recent violent ideation or behavior). No participants were ineligible due to a history of pre-existing anti-social behavior. Of the seven eligible prospective participants, six agreed to participate and provided written informed consent; one prospective participant declined to provide consent due to concerns about confidentiality. EIS participants also provided consent for research staff to speak to their primary clinician for additional information, such as their date of first symptoms, length of treatment, and diagnosis. No EIS participants ended the interview early or skipped questions. All screened EIS staff (*n* = 12) were eligible.

### Measurement and Procedures

**Fig. 1:**
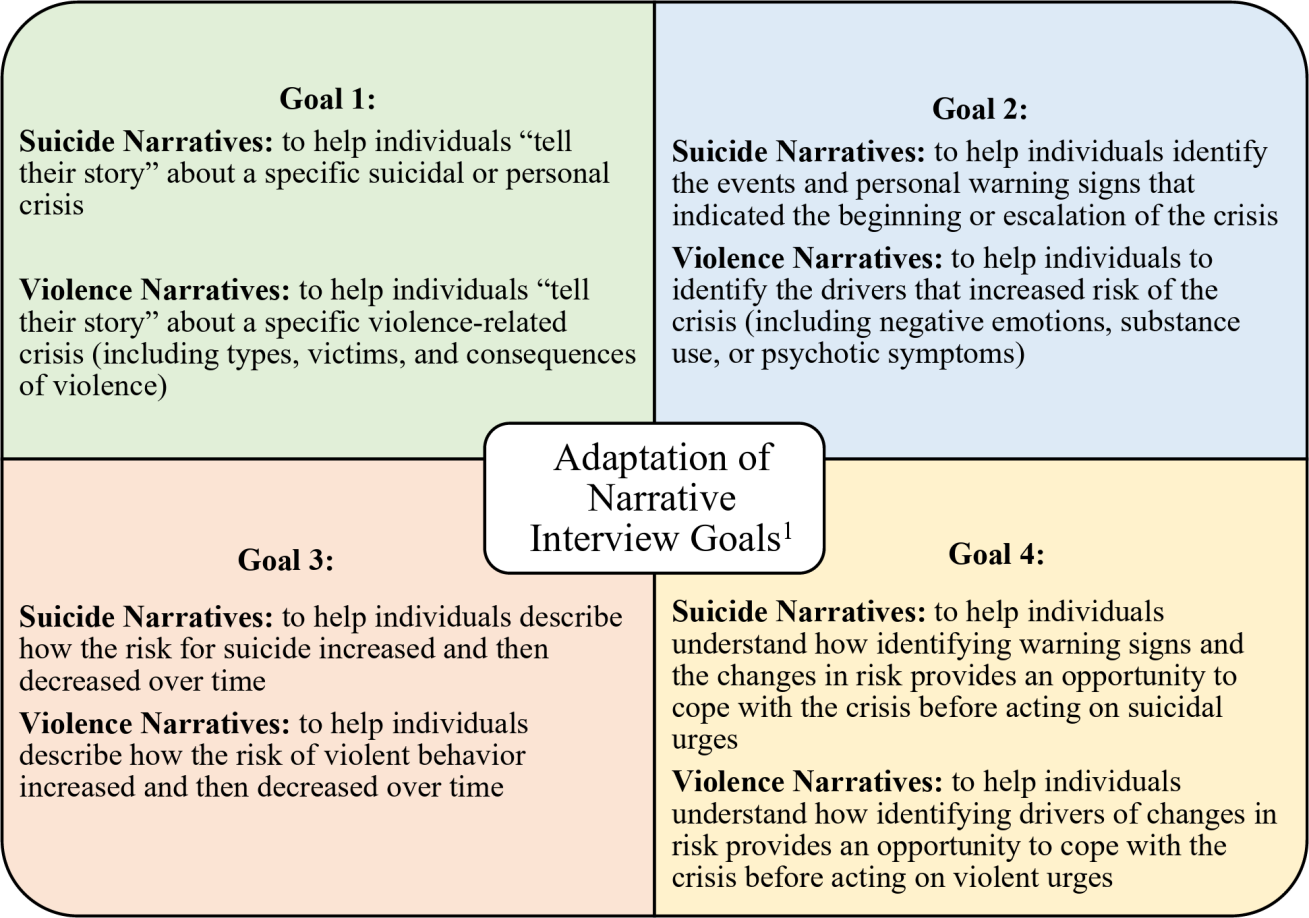
Adaptation of narrative interview goals. ^1^ All suicide narrative goals adapted from Barbara Stanley’s presentation at the “Substance Use and Mental Health Services Administration” presentation on August 20, 2019

The “suicide narrative” format from the Stanley-Brown Safety Planning Intervention (SPI) served as the basis for comparisons drawn in this study. The SPI is an evidence-based suicide prevention intervention that can be used to develop a suicide narrative to comprehensively analyze the factors that led a patient to consider ending their life (Stanley et al., 2018). For this project, the interview guide sought to develop “violence narratives” by asking EIS participants detailed questions about a specific episode of violent ideation or behavior. We used a similar method to debrief an episode of violent ideation or behavior with EIS staff. First, violent ideation or behavior was defined using the CIS-V (described above). Once an instance of violent ideation or behavior was identified, detailed questions were asked about the circumstances leading up to the episode, the emotional state of persons involved in the episode, and the consequences of the episode. A semi-structured interview guide was developed and pilot-tested, with revisions for clarity and flow before data collection. As with the suicide narrative process, EIS participants and EIS staff were asked questions to assist them in telling their story regarding a specific instance of violent ideation or behavior (see Fig. 1 for adaptation process). These included (but were not limited to): What was going on earlier that day? What were you doing? What were you thinking when this was happening? What were you feeling? Did anything change for you that led to this happening? Were you hurt in what happened? Has it affected your family? Your housing?

Per clinic policy, EIS clinicians did not receive compensation for their participation in this study, as interviews were completed during their salaried workday. EIS peer specialists were offered $50 compensation when in accordance with their clinic policy. EIS participants received $50 compensation for participating in the interview. All study procedures were initially approved by the New York State Psychiatric Institute IRB (Protocol #8211) and subsequently approved by the Columbia University Irving Medical Center IRB (Protocol #AAAU8609).

### Data Analyses

Interviews were audio recorded and professionally transcribed. Data analysis was conducted concurrently with data collection using an iterative approach. Interviews were analyzed as they were completed, allowing emerging themes to inform subsequent interviews. First, two trained members of the research team (XXX and YY) independently read transcripts for familiarity with content. Transcripts were then systematically analyzed to identify relevant details and patterns of violent episodes, including those that aligned with domains of the FVT (Rudd, 2006). Both deductive and inductive approaches were used. First, we identified general thematic domains regarding types of violence as defined by the CIS-V. These domains included drivers of violence, types of violence, victims of violence, consequences of violence, and protective factors against violence. Then, XXX and YY read a sample of interview transcripts and used open coding to create an initial list of codes to categorize the data. Codes were developed iteratively through review of subsequent transcripts until a final codebook was produced. Texts were then subjected to systematic line-by-line coding in Dedoose (Dedoose Version 9.0.17). The domains and codes are provided in the Appendix. During this process, credibility was enhanced through “peer debriefing” with a clinical mentorship team and “member checks” with the OnTrackNY central leadership, who provided feedback on data interpretation. To enhance confirmability, the coders strived to bracket their biases with reflexive practice (Jacoby, 2017; Tufford & Newman, 2012). For example, during data collection, the interview included questions on personal connections to and beliefs about violence. During data analysis, the coders discussed this section of the interview together and reflected on how their own experiences could influence data interpretation.

## Results

The demographic characteristics of each research participant are displayed in Table 1. Most EIS participants were male (*n* = 4, 66.7%), black (*n* = 4, 66.7%), and not Hispanic or Latino (*n* = 4, 66.7%). Most EIS staff were female (*n* = 10, 83.3%), white (*n* = 10, 83.3%), and not Hispanic or Latino (*n* = 11, 91.7%). The average age was 21 years among EIS participants (SD = 1.4) and 45.5 years among EIS staff (SD = 10.4). Overall, EIS participants who participated were willing to discuss prior violence in detail. Similarly, EIS staff had multiple examples of violence to draw from and were able to narrate episodes of participant violence in detail.

**Table 1:** Demographic characteristics of interviewees

		EIS participants (*N* = 6) *N* (%)	EIS staff (*N* = 12) *N* (%)
Age (mean [SD)		21 (1.4)	45.5 (10.4)
Male		4 (66.7)	2 (16.7)
Race			
White		0 (0)	10 (83.3)
Black		4 (66.7)	2 (16.7)
American Indian/Alaska Native		1 (16.7)	0 (0)
Multiracial		1 (16.7)	0 (0)
Ethnicity			
Hispanic/Latino		2 (33.3)	1 (8.3)

### Violence Narratives

The adapted narrative format was a feasible method to elucidate details about prior violent episodes, yielding rich descriptions of the circumstances surrounding psychosis-related violence. These instances included both threats and acts of violence, which were rarely premeditated. Types of violence discussed by study participants included ideation, threats, and acts related to hitting others or throwing objects, arson, child endangerment, and erratic driving. Examples that demonstrate the breadth of the violence discussed include:

“I just want to beat [my family] up. I’m not going to, but I just feel like I want to.” [Participant #5].

“I just heard a voice that said ‘take the car,’ and I drove for hours at that point… I was just crying, and I was angry and then I almost killed myself like three times… I was doing 120 [mph] down the back road… the third time was when I crashed [intentionally into a neighbor’s home].” [Participant #6].

“When his first episode started to happen, largely in the form of paranoia, he felt like people were following him, and I think there were some voices… the symptoms intensified, and it was really out of fear… he got so scared that he set the hotel on fire.” [Clinician #5].

### Victims of Violence

Victims of violence were varied but often included family members (especially parents). Other victims included friends, intimate partners, healthcare workers, law enforcement officers, animals, and strangers.

“[His] dad just had a triple bypass and was the one who brought him to the emergency room. Well, 90 minutes after [the patient] was discharged, he punched his father in the chest” [Clinician #3].

“One of my patients fractured their mother’s eye socket, thinking she was the devil… somebody assaulted a dog before, that was a tough one, I have to say. He just arbitrarily punched the dog” [Clinician #3].

In general, EIS participants reported that violence was often directed toward persons in close proximity at times of conflict, especially family members. One participant said, “My brother was just being mean and stuff like that, just being annoying… I just wanted to beat him up.” [Participant #4] Similarly, another participant described how a physical conflict with their mother arose after they took her car, explaining, “I end up fighting my mom physically… I blacked out and we started fighting, and then it ended with me putting my mom in a headlock.” [Participant #6].

### Consequences of Violence

The consequences of violence often included negative impacts on emotional wellbeing, personal stability, and engagement in treatment. Participants reported that these repercussions sometimes resulted in the termination of work and educational opportunities, periods of homelessness, and incarceration resulting from legal charges.

#### Emotional Wellbeing

EIS participants reported that mental health was negatively affected by violent behavior. Specific feelings that were shared included sadness, anger, and confusion. One participant said, “I didn’t want to do it. I wanted it out of my mind, but it was more or less pissing me off because I know I don’t want this thought here.” [Participant #1].

The psychological health of family members was also affected by violent behavior, with family members most often described as fearful. One peer specialist shared:

“The participant has a younger brother, I’m guessing between the ages of five and seven. His mother has reported to me that he [the younger brother] does not feel safe being in the house, and that he is afraid of his older brother. I think at this point the entire family is pretty scared of him and the things that he’s going through.” [Peer Specialist #4].

#### Personal Stability

Many of consequences of violence were caused by the deterioration of existing personal relationships, especially with family members who previously provided financial or housing support for EIS participants. When describing the effects of their violent behavior, one participant recalled, “My mom ended up pressing charges on me, and I couldn’t go home because she put a protective order against me, and then after that [I] was homeless [for] six months.” [Participant #6].

When describing a similar situation faced by another participant, one peer specialist shared the following:

“[He faced] eviction, so it did affect them in that aspect, because of the money issues… so we’re trying to help them get housing or get in a community setting where they can have different resources that we can go to… unfortunately, his mother had kicked him out. [She] had referred him to a shelter, but he didn’t go, [and he wasn’t] taking care of his hygiene. [He] tried to go back home [but] wasn’t allowed in… he’s basically homeless right now. He’s going from home to home.” [Peer Specialist #5].

#### Engagement in Treatment

Participants who had engaged in violence had varying reactions to subsequent treatments. One clinician reported that their participant seemed more willing to receive treatment after his violent episode:

“We had a lot more contact, [and] he got started on an injection once again… I think that experience was something that was really scary to think about, and he was like, “That’s not who I am as a person, it’s so sad to think that I could think that way or want to do something like that.” So I think a lot of more positive things came from that [violent episode] than negative.” [Clinician #4].

In contrast, however, others felt that violence could diminish a participant’s willingness to receive treatment if they denied that their behavior was violent.

“The biggest barrier would be if they don’t identify that they actually did it [a violent act]. That would be tough because usually patients have some recollection of something they’ve done, but if they’re in complete and utter denial, [saying] “Oh no, I didn’t do that,” that’s going to be a big barrier.” [Clinician #3].

### Drivers of Violence

#### The Cognitive System

In the FVT model, the first of the potentially aggravating set of factors involves the cognitive system, which encompasses information processing, such as selection of data, attentional process, memory, and subsequent recall (Rudd, 2006). Cognitive processes may be affected by symptoms of psychosis, which in our analyses of drivers of violence, most often included auditory hallucinations and delusions (Arciniegas, 2015). For example, one participant described a recent episode of violence triggered by auditory hallucinations:

“I was sitting down watching TV. The voices in my head started speaking… they’re talking about.

people’s looks, they start saying people were ugly. Now I try to fix it by saying the opposite of them [but] they are throwing temper tantrums. That’s what triggered my anger, so I started to rampage around the house.” [Participant #2].

Another participant recalled that how auditory hallucinations (re-triggered by memories of childhood sexual trauma) often preceded their aggressive behavior:

“Around that time, I was hearing voices again… whatever I had in my past, like childhood stuff about me getting raped triggered me, and I thought I heard [the voices talking about it]. I was just feeling a whole bunch of anger, and that’s when I started speeding [in the car]… I called my mom, [and I was trying to tell her] I’m going to kill myself. And then she just yelled… I was calm but as soon as she yelled, it triggered me right back to anger. That’s when I just drove into the balcony.” [Participant #6].

EIS staff similarly agreed that symptoms of psychosis seemed temporally linked to violent behavior, with one peer specialist recalling: “He seemed like he was deep in psychosis, but he denied hearing voices. He’s also experiencing high levels of mania. I think that those high levels of mania can trigger some kind of voices that might have told him to hurt other people.” [Peer Specialist #4].

#### The Affective System

Under the FVT model, the affective system encompasses emotional states that influence behavior (Rudd, 2006). Examples include stress responses, impulses and moods that shape how a person thinks, feels and behaves (Gross et al., 2019). In the violence narratives, EIS participants often described feelings of anger when dealing with personal conflict or tense situations.

“[It made me] mostly react in an angry way, like wanting to fight someone. But there was no one to fight, it was me saying it to myself.” [Participant #3].

“My stressor sometimes is [when] I feel like my family won’t tell me the truth and stuff, and then I just lose it.” [Participant #5].

Another participant shared, “My psychosis triggered, and it triggered my anger as well. I was punching holes in the wall, throwing stuff, I even threw my old phone.” [Participant #2].

In addition to anger, fear was a common affective state preceding episodes of violence.

“His mother called for an ambulance and the police came, as they do always if you call for an ambulance. He got really scared and he ran away, and they chased him, and they tackled him, and one of the police officers got some scratches in the process. He was charged with assaulting a police officer, which is a felony.” [Clinician #2].

EIS staff members shared similar concerns about anger and fear driving violence among EIS participants. One staff member shared how an EIS participant would voice violent thoughts in the setting of anger and fear over their brother’s unsolved murder.

“[He] was being very suspicious, talking continuously about getting revenge on the person that killed his brother several years ago. Just verbally speaking violently about what he would do, the old neighborhood he would go back to, go up and beat them up, because they still haven’t [found] the person that fatally killed his brother.” [Peer Specialist #2].

#### The Physiological System

According to the FVT model, the physiological system consists of the bodily processes that can orient an individual for fight or flight behaviors (Rudd, 2006). One participant described notable changes to their diet and sleep patterns prior to an episode of violent behavior: “Before I crashed the car, I didn’t eat or sleep for like two days,…before that I was just suppressing every single emotion. So, I just went completely numb after a certain point, and then it just came out.” [Participant #6].

Another EIS participant described the physical sensations that preceded an instance of violent ideation, sharing, “It was very sudden. It was a short-lived experience… it kind of flushed through my whole body and as it hit me, I was like, ‘My mom’s not here, so I’m not gonna cut her throat right now. That’s not even possible.’” [Participant #1].

#### The Behavioral System

The behavioral system is the broadest of the four FVT domains, and includes variables such as self-soothing strategies, interpersonal interactions, and substance use (Rudd, 2006; Rugo-Cook et al., 2021). The most commonly discussed risk factor for violence from the behavioral system was substance use. One EIS participant stated that they now avoid substances after being hospitalized repeatedly because of substance use, reflecting, “I kept coming here [the hospital], kept doing the groups just to get away for a couple hours. I would go on walks; I would smoke blacks [amphetamines]… I stopped drinking and all that stuff after a while.” [Participant #2].

EIS staff also expressed belief that substance use drove violent behavior. When discussing the role of alcohol use, an EIS clinician shared, “Definitely substance use does play some kind of role, although it’s not so obvious… I think, you know, you see a little bit more like ‘I got drunk and had a fight’ with alcohol.” [Clinician #2] Another clinician reflected that when participants choose to self-medicate with marijuana, they felt that these efforts could worsen paranoia, saying, “I think across the board, there’s a lot of medicating with marijuana, and that’s certainly enhancing paranoia that’s already there.” [Clinician #5] Another peer specialist shared their view of the relationship between cannabis and violence, stating, “Absolutely. They’re definitely connected. Typically, even with our participants, most of them have cannabis use, [and it] affects their hallucinations, their delusions, it definitely impacts it.” [Peer Specialist #3].

## Discussion

Existing research about violence among young adults with early psychosis has identified several risk factors for violent behavior, including increased impulsivity, hostility, and substance use (Large & Nielssen, 2011; Rolin et al., 2019, 2024a, b). In addition, treatment targeting anger and symptoms of psychosis has demonstrated efficacy in reducing violent behavior in a similar population (Haddock et al., 2009; Rolin et al., 2024a, b). Given the lack of literature that highlights the perspective of young adults with early psychosis on this topic, the aim of this study was to demonstrate the feasibility of the narrative format and to deepen the field’s understanding of their lived experiences of violent ideation or behavior through application of the FVT.

To the authors’ knowledge, this is the first study seeking to utilize suicide narratives, a technique that has been used to identify target mechanisms in suicide prevention research, to understand violence (Adler et al., 2020; Chesin et al., 2018). Overall, this study found that it was feasible to help individuals share information about a specific violence-related crisis by eliciting violence narratives from young adults with early psychosis and their treatment teams. Other studies exploring violence in psychosis have drawn similar conclusions through discussions of clinical vignettes (Lambe et al., 2024; Whiting et al., 2024). In our study, we were able to demonstrate that most EIS participants were both willing and able to discuss their own prior violence in detail, answering all questions and participating in the full interview.

An important outcome from the “violence narratives” was the identification of suffering as a significant impact of violent behavior, with participants and their treatment teams noting poor emotional wellbeing and strains on existing personal and clinical relationships. EIS participants and staff shared how violence, even a first violent act, had significant negative impacts on young people’s lives, leading to homelessness, lost work and educational opportunities, and incarceration. Overall, these interviews spoke to how devastating even a single violent act could be for an EIS participant. This is important to note, because while most people with early psychosis do not act violently, those who do tend to experience major disruptions in their lives. The authors hope that these narratives can lead to more compassionate and therapeutic understandings of violence for young adults with early psychosis, more analogous to the “psychache” of negative emotions (despair; fear; sadness; guilt; shame) that has been described for people with suicidal behavior, rather than reflexively labeling violent behavior as simply “bad”(Madeira & Miranda, 2021).

**Fig. 2:**
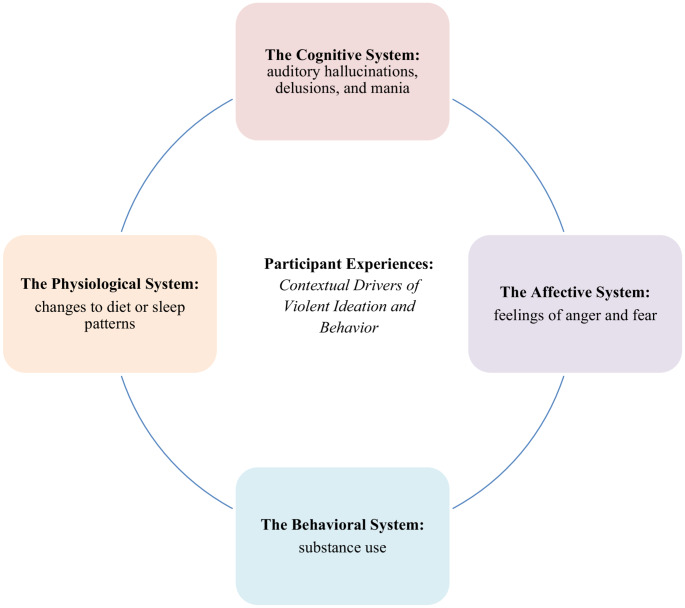
Drivers of violence assessed through the fluid vulnerability theory

In addition, we used the FVT to examine near-term risk factors in these violence narratives from a patient-centered perspective. In this study, EIS participants and staff identified a number of risk factors from the FVT domains that could spark either violent ideation or behavior, thus mirroring the concept of the suicidal mode in the FVT. Symptoms of psychosis (the cognitive system), feelings of anger and fear (the affective system), changes to diet or sleep patterns (the physiological system), and substance use (the behavioral system) were mentioned as important factors in the timeline of developing violent behavior, which was largely impulsive and not premeditated (Fig. 2). These near-term risk factors were repeatedly suggested as key drivers in violent ideation by EIS participants and staff, an area of notable concurrence between all groups of interview participants. These findings are supported by existing research regarding risk factors associated with violence among persons with psychosis (Moulin et al., 2018, 2022).

### Limitations

While the results of these interviews show promise in incorporating violence narratives in EIS treatment, several limitations exist in this study. Interviewer presence may have affected subject behavior, which is an inherent limitation of qualitative research, as both participants and staff members may have felt pressured to answer in more socially desirable ways. The participant sample in this study also only consisted of young adults with early psychosis receiving ongoing treatment at one EIS clinic, which may limit generalizability, given that participants lived in the same geographic area. Therefore, this study’s findings may not be generalizable to programs outside of OnTrackNY. Lastly, participant viewpoints and experiences may differ from young adults with early psychosis who have not received treatment. Despite these limitations, the perspectives yielded in this study still offer valuable insight into experiences with violence that may only be attainable through qualitative research techniques.

### Future Research Directions

This project demonstrates the feasibility of conducting research about violence in EIS settings. In addition to identifying potential target mechanisms for future interventions through the lens of the FVT, the authors hope that these narratives can lead to more nuanced understandings of violence for young adults with early psychosis. Our results suggest there is an urgent need for preventive interventions for violence for young adults with early psychosis, as both staff and patient participants outlined the long-term impacts of violent behavior. Using the framework of the FVT, we categorized valuable information about key drivers of violence including symptoms of psychosis, feelings of anger and fear, changes to physiological processes, and substance use. These near-term risk factors were repeatedly suggested as important factors in the development of violence by EIS participants, clinicians, and peer specialists, an area of notable concurrence between the groups of interview participants. These drivers could be the target of future research in the development and testing of preventive interventions for violence.

## Conclusions

This study demonstrates the feasibility of using narrative interviews to explore violent behavior among young adults with early psychosis. By applying the FVT, we identified key near-term risk factors and highlighted the emotional and social consequences of violence. These findings lay the foundation for future prevention interventions grounded in patient-centered perspectives and trauma-informed care.

## Data Availability

No datasets were generated or analysed during the current study.

## Appendix. Coded categories based on interview transcripts

**Table:**

Domain	Code
Drivers of violence	Social stressor
	Poor support system
	Psychosis
	Anger/hostility
	Substance use
	Trauma
Types of violence	Physical force to have sex
	Child endangerment
	Erratic driving
	Hit with first, object, or beat someone up
	Kicked/bitten/choked
	Arson
	Property damage
	Pushed/grabbed/shoved
	Slapped someone
	Threated or used knife, gun, or lethal weapon
	Thrown something
Victims of violence	Intimate partner
	Friend/acquaintance
	Healthcare worker
	Close non-parent family member
	Law enforcement
	Parent
	Stranger
Consequences of violence	Injuries
	Distress
	Gun ownership
	Impact on housing
	Impact on work/school
	ACS involvement
	Impact on relationships
	Legal consequences
Protective factors against violence	Good social support
	Love

## References

[CR1] Adams, S. W., & Yanos, P. T. (2020). Pathways to aggression and violence in psychosis without longstanding antisocial behavior: A review and proposed psychosocial model for integrative clinical interventions. *Psychiatry Research*, *293*, 113427. 10.1016/j.psychres.2020.113427 32866792 10.1016/j.psychres.2020.113427

[CR2] Adler, A., Jager-Hyman, S., Brown, G. K., Singh, T., Chaudhury, S., Ghahramanlou-Holloway, M., & Stanley, B. (2020). A qualitative investigation of barriers to seeking treatment for suicidal thoughts and behaviors among army soldiers with a deployment history. *Archives of Suicide Research: Official Journal of the International Academy for Suicide Research,**24*(2), 251–268. 10.1080/13811118.2019.1624666 31237808 10.1080/13811118.2019.1624666

[CR3] Ammerman, B. A., Kleiman, E. M., Uyeji, L. L., Knorr, A. C., & McCloskey, M. S. (2015). Suicidal and violent behavior: The role of anger, emotion dysregulation, and impulsivity. *Personality and Individual Differences*, *79*, 57–62. 10.1016/j.paid.2015.01.044

[CR4] B Arciniegas, D. (2015). Psychosis. *Continuum (Minneap Minn)*, *21*(3 Behavioral Neurology and Neuropsychiatry), 715–736. 10.1212/01.CON.0000466662.89908.e7 26039850 10.1212/01.CON.0000466662.89908.e7 PMC4455840

[CR5] Bello, I., Lee, R., Malinovsky, I., Watkins, L., Nossel, I., Smith, T., Ngo, H., Birnbaum, M., Marino, L., Sederer, L. I., Radigan, M., Gu, G., Essock, S., & Dixon, L. B. (2017). OnTrackNY: The development of a coordinated specialty care program for individuals experiencing early psychosis. *Psychiatric Services,**68*(4), 318–320. 10.1176/appi.ps.201600512 27973999 10.1176/appi.ps.201600512 PMC5846122

[CR6] Chesin, M. S., Brodsky, B. S., Beeler, B., Benjamin-Phillips, C. A., Taghavi, I., & Stanley, B. (2018). Perceptions of adjunctive mindfulness-based cognitive therapy to prevent suicidal behavior among high suicide-risk outpatient participants. *Crisis,**39*(6), 451–460. 10.1027/0227-5910/a000519 29848083 10.1027/0227-5910/a000519

[CR7] Dixon, L. B., Goldman, H. H., Bennett, M. E., Wang, Y., McNamara, K. A., Mendon, S. J., Goldstein, A. B., Choi, C. W., Lee, R. J., Lieberman, J. A., & Essock, S. M. (2015). Implementing coordinated specialty care for early psychosis: The RAISE connection program. *Psychiatric Services,**66*(7), 691–698. 10.1176/appi.ps.201400281 25772764 10.1176/appi.ps.201400281 PMC5637730

[CR8] Farrell, C., & Zimmerman, G. M. (2019). Violent lives: Pathways linking exposure to violence to suicidal behavior in a national sample. *Archives of Suicide Research: Official Journal of the International Academy for Suicide Research,**23*(1), 100–121. 10.1080/13811118.2017.1404517 29220611 10.1080/13811118.2017.1404517

[CR9] First, M., Williams, J., Karg, R., & Spitzer, R. (2015). *Structured clinical interview for DSM-5-clinical version (SCID-5 for DSM-5, clinical version; SCID-5-CV, version 1.0. 0)*. American Psychiatric Association.

[CR10] Fusar-Poli, P., McGorry, P. D., & Kane, J. M. (2017). Improving outcomes of first-episode psychosis: An overview. *World Psychiatry,**16*(3), 251–265. 10.1002/wps.20446 28941089 10.1002/wps.20446 PMC5608829

[CR11] Gross, J. J., Uusberg, H., & Uusberg, A. (2019). Mental illness and well-being: An affect regulation perspective. *World Psychiatry,**18*(2), 130–139. 10.1002/wps.20618 31059626 10.1002/wps.20618 PMC6502417

[CR12] Haddock, G., Barrowclough, C., Shaw, J. J., Dunn, G., Novaco, R. W., & Tarrier, N. (2009). Cognitive-behavioural therapy v. social activity therapy for people with psychosis and a history of violence: Randomised controlled trial. *British Journal of Psychiatry,**194*(2), 152–157. 10.1192/bjp.bp.107.039859 10.1192/bjp.bp.107.039859 19182178

[CR13] Huang, X., King, C., & McAtee, J. (2018). Exposure to violence, neighborhood context, and health-related outcomes in low-income urban mothers. *Health & Place,**54*, 138–148. 10.1016/j.healthplace.2018.09.008 30265943 10.1016/j.healthplace.2018.09.008

[CR14] Hvidkjaer, K. L., Ranning, A., Madsen, T., Fleischer, E., Eckardt, J. P., Hjorthøj, C., Cerel, J., Nordentoft, M., & Erlangsen, A. (2021). People exposed to suicide attempts: Frequency, impact, and the support received. *Suicide and Life-Threatening Behavior,**51*(3), 467–477. 10.1111/sltb.12720 33258173 10.1111/sltb.12720

[CR15] Jacoby, S. F. (2017). The insight and challenge of reflexive practice in an ethnographic study of black traumatically injured patients in Philadelphia. *Nursing Inquiry*. 10.1111/nin.12172 27862663 10.1111/nin.12172 PMC5432420

[CR16] Jokinen, J., Forslund, K., Ahnemark, E., Gustavsson, J. P., Nordström, P., & Asberg, M. (2010). Karolinska interpersonal violence scale predicts suicide in suicide attempters. *Journal of Clinical Psychiatry*, *71*(8), 1025–1032. 10.4088/JCP.09m05944blu 20797380 10.4088/JCP.09m05944blu

[CR17] Lambe, S., Cooper, K., Fazel, S., & Freeman, D. (2024). Psychological framework to understand interpersonal violence by forensic patients with psychosis. *British Journal of Psychiatry*, *224*(2), 47–54. 10.1192/bjp.2023.132 10.1192/bjp.2023.132 PMC10807973 37861077

[CR18] Large, M. M., & Nielssen, O. (2011). Violence in first-episode psychosis: A systematic review and meta-analysis. *Schizophrenia Research*, *125*(2–3), 209–220. 10.1016/j.schres.2010.11.026 21208783 10.1016/j.schres.2010.11.026

[CR19] Madeira, L., & Miranda, A. (2021). A narrative review of suicide: Aiming at a more encompassing understanding. *Philosophies,**6*, Article 74. 10.3390/philosophies6030074

[CR20] Masucci, M. D., Wall, M. M., Califano, A., Hesson, H., Brucato, G., Cohen-Romano, C., Bornico, M., Sommerich, K., Provenzano, F. A., Snyder, C., Lieberman, J. A., Appelbaum, P. S., & Girgis R. R. (n.d.). Phenomenology of violent ideation in individuals at clinical high-risk for psychosis *Unpublished*.

[CR21] Moran, M. (2019). Affective psychosis common among first-episode patients. *Psychiatric News,**54*(22), null. 10.1176/appi.pn.2019.11b20

[CR22] Moran, P., & Hodgins, S. (2004). The correlates of comorbid antisocial personality disorder in schizophrenia. *Schizophrenia Bulletin*, *30*(4), 791–802. 10.1093/oxfordjournals.schbul.a007132 15954191 10.1093/oxfordjournals.schbul.a007132

[CR23] Moulin, V., Golay, P., Palix, J., Baumann, P. S., Gholamrezaee, M. M., Azzola, A., Gasser, J., Do, K. Q., Alameda, L., & Conus, P. (2018). Impulsivity in early psychosis: A complex link with violent behaviour and a target for intervention. *European Psychiatry: The Journal of the Association of European Psychiatrists*, *49*, 30–36. 10.1016/j.eurpsy.2017.12.003 29353178 10.1016/j.eurpsy.2017.12.003

[CR24] Moulin, V., Framorando, D., Gasser, J., & Dan-Glauser, E. (2022). The link between cannabis use and violent behavior in the early phase of psychosis: The potential role of impulsivity. *Frontiers in Psychiatry,**13*, 746287. 10.3389/fpsyt.2022.746287 35392388 10.3389/fpsyt.2022.746287 PMC8980530

[CR25] Mueser, K. T., Crocker, A. G., Frisman, L. B., Drake, R. E., Covell, N. H., & Essock, S. M. (2006). Conduct disorder and antisocial personality disorder in persons with severe psychiatric and substance use disorders. *Schizophrenia Bulletin,**32*(4), 626–636. 10.1093/schbul/sbj068 16574783 10.1093/schbul/sbj068 PMC2632266

[CR26] Purcell, R. (2015). *Clinical practice in youth mental health: Assessing and managing risk of violence in early psychosis*. Orygen. Issue. https://www.orygen.org.au/Training/Resources/Psychosis/Clinical-practice-points/Assessing-and-managing-risk-of-violence-in-EP/Assessing-and-managing-risk-of-violence-in-early-p?ext=.#:~:text=Dynamic/20risk/20factors/20can/20change,violence/20during/20the/20short-term.%26;text=Static/20risk/20factors/20can’t,longer-term/20risk/20for/20violence.%26;text=Static/20risk/20factors/20can/20indicate,explore/20specific/20areas/20of/20risk

[CR27] Rolin, S. A., Marino, L. A., Pope, L. G., Compton, M. T., Lee, R. J., Rosenfeld, B., Rotter, M., Nossel, I., & Dixon, L. (2019). Recent violence and legal involvement among young adults with early psychosis enrolled in coordinated specialty care. *Early Intervention in Psychiatry,**13*(4), 832–840. 10.1111/eip.12675 29740953 10.1111/eip.12675 PMC6226380

[CR28] Rolin, S. A., Caffrey, D., Flores, M. G., Mootz, J., Bello, I., Nossel, I., Compton, M. T., Stanley, B., Wainberg, M. L., Dixon, L. B., Appelbaum, P. S., & Pope, L. G. (2024a). Qualitative evaluation of acceptability and feasibility of a behavioral intervention to reduce violence among young adults with early psychosis. *Community Mental Health Journal*. 10.1007/s10597-024-01343-x 39172311 10.1007/s10597-024-01343-x PMC11703671

[CR29] Rolin, S. A., Caffrey, D., Flores, M. G., Pope, L. G., Mootz, J., Bello, I., Nossel, I., Compton, M. T., Stanley, B., Wainberg, M., Dixon, L. B., & Appelbaum, P. S. (2024b). An open pilot trial of a behavioural intervention to reduce violence by young adults with early psychosis receiving treatment in an early intervention services setting: A protocol. *Early Intervention in Psychiatry*. 10.1111/eip.13543 38705578 10.1111/eip.13543 PMC11535252

[CR30] Rudd, M. D. (2006). Fluid vulnerability theory: A cognitive approach to understanding the process of acute and chronic suicide risk. In *Cognition and suicide: Theory, research, and therapy.* (pp. 355–368). American Psychological Association. 10.1037/11377-016

[CR31] Rugo-Cook, K. F., Kerig, P. K., Crowell, S. E., & Bryan, C. J. (2021). Fluid vulnerability theory as a framework for understanding the association between posttraumatic stress disorder and suicide: A narrative review. *Journal of Traumatic Stress,**34*(6), 1080–1098. 10.1002/jts.22782 34881461 10.1002/jts.22782

[CR32] Stanley, B., & Brown, G. K. (2019). The safety planning intervention to reduce suicide risk for people with SM. Substance abuse and mental health services administration.

[CR33] Stanley, B., Brown, G. K., Brenner, L. A., Galfalvy, H. C., Currier, G. W., Knox, K. L., Chaudhury, S. R., Bush, A. L., & Green, K. L. (2018). Comparison of the safety planning intervention with follow-up vs usual care of suicidal patients treated in the emergency department. *JAMA Psychiatry,**75*(9), 894–900. 10.1001/jamapsychiatry.2018.1776 29998307 10.1001/jamapsychiatry.2018.1776 PMC6142908

[CR34] Swanson, J. W., Swartz, M. S., Van Dorn, R. A., Volavka, J., Monahan, J., Stroup, T. S., McEvoy, J. P., Wagner, H. R., Elbogen, E. B., & Lieberman, J. A. (2008). Comparison of antipsychotic medication effects on reducing violence in people with schizophrenia. *British Journal of Psychiatry*, *193*(1), 37–43. 10.1192/bjp.bp.107.042630 10.1192/bjp.bp.107.042630 PMC2801826 18700216

[CR35] Tufford, L., & Newman, P. (2012). Bracketing in qualitative research. *Qualitative Social Work*, *11*(1), 80–96. 10.1177/1473325010368316

[CR36] Volavka, J., & Citrome, L. (2011). Pathways to aggression in schizophrenia affect results of treatment. *Schizophrenia Bulletin*, *37*(5), 921–929. 10.1093/schbul/sbr041 21562140 10.1093/schbul/sbr041 PMC3160235

[CR37] Whiting, D., Glogowska, M., Fazel, S., & Lennox, B. (2024). Approaches and challenges to assessing risk of violence in first episode psychosis: A qualitative interview study of clinicians, patients and carers. *Early Intervention in Psychiatry*. 10.1111/eip.13502 38356414 10.1111/eip.13502 PMC7618059

[CR38] Wolford-Clevenger, C., & Smith, P. N. (2017). The conditional indirect effects of suicide attempt history and psychiatric symptoms on the association between intimate partner violence and suicide ideation. *Personality and Individual Differences,**106*, 46–51. 10.1016/j.paid.2016.10.042 29056805 10.1016/j.paid.2016.10.042 PMC5647881

